# Long-Term Nitrogen Addition Eliminates the Cooling Effect on Climate in a Temperate Peatland

**DOI:** 10.3390/plants14081183

**Published:** 2025-04-10

**Authors:** Fan Lu, Boli Yi, Kai Qin, Zhao-Jun Bu

**Affiliations:** 1 Jiangsu Key Laboratory of Coal-Based Greenhouse Gas Control and Utilization, School of Environment and Spatial Informatics, China University of Mining and Technology, Xuzhou 221116, China; luf785@cumt.edu.cn (F.L.); qinkai@cumt.edu.cn (K.Q.); 2 Jilin Provincial Key Laboratory for Wetland Ecological Processes and Environmental Change in the Changbai Mountains, Institute for Peat and Mire Research, Renmin 5268, Changchun 130024, China; ybl@imufe.edu.cn; 3 School of Statistics and Mathematics, Inner Mongolia University of Finance and Economics, No. 185 Bei Erhuan Road, Hohhot 010051, China; 4 Key Laboratory of Geographical Processes and Ecological Security in Changbai Mountains, Ministry of Education, School of Geographical Sciences, Northeast Normal University, Renmin 5268, Changchun 130024, China

**Keywords:** peat decomposition, extracellular enzyme activity, CO_2_, *Sphagnum*, methane

## Abstract

Peatlands play a crucial role in global carbon (C) sequestration, but their response to long-term nitrogen (N) deposition remains uncertain. This study investigates the effects of 12 years of simulated N addition on CO_2_ and CH_4_ fluxes in a temperate peatland through in situ monitoring. The results demonstrate that long-term N addition significantly reduces net ecosystem exchange (NEE), shifting the peatland from a C sink to a C source. This transition is primarily driven by a decline in aboveground plant productivity, as *Sphagnum* mosses were suppressed and even experienced mortality, while graminoid plants thrived under elevated N conditions. Although graminoid cover increased, it did not compensate for the GPP loss caused by *Sphagnum* decline. Instead, it further increased CH_4_ emissions. These findings suggest that sustained N input may diminish the C sequestration function of peatlands, significantly weakening their global cooling effect.

## 1. Introduction

Peatlands cover only about 3% of the global land area, but store about ~600 Gt of the global soil C and play a vital role in the global carbon (C) cycle [1,2]. A peatland is both a C sink, which can effectively mitigate climate change, and a C source, which can exacerbate global warming under certain conditions [3,4,5,6]. However, the C balance of peatlands is highly susceptible to environmental changes, especially the significant increase in nitrogen (N) deposition caused by anthropogenic activities [7]. Over the past 150 years, the amount of reactive N in the atmosphere has increased more than 10-fold, driven by agricultural fertilization, fossil fuel combustion, and industrial activities, and global N deposition levels are expected to increase another 2–3 times by 2050 [8,9,10,11]. This anthropogenically driven N enrichment has profound impacts on nutrient-limited peatland ecosystems, potentially not only weakening C storage capacity but also significantly altering greenhouse gas emission fluxes [12,13,14].

Peatland ecosystems are extremely sensitive to N deposition, which is closely related to the characteristics of their dominant plant—*Sphagnum* moss, which absorbs atmospheric N through its capitulum branches [15]. N addition affects the production and consumption of greenhouse gases (CO_2_ and CH_4_) by regulating plant and microbial activity and changing related biogeochemical reactions. In most ecosystems, N addition generally increases plant growth, thereby increasing C storage in plant biomass [16,17,18,19]. However, this positive effect is less common in non-forest ecosystems [14,20]. In peatlands, N addition may inhibit the growth of *Sphagnum* mosses or even cause their death, significantly reducing net ecosystem exchange (NEE) [7,14]. At the same time, increased N deposition generally promotes the growth of vascular plants [21,22]. Although the expansion of vascular plants may increase carbon absorption capacity by enhancing photosynthesis in the short term, the increase in vegetation N content will also accelerate the decomposition of litter [23,24,25,26], leading to higher CO_2_ emissions. In addition, studies have shown that higher levels of atmospheric N deposition may further exacerbate peatland C loss by enhancing heterotrophic respiration and dissolved organic carbon (DOC) leaching [12]. Therefore, the promotion of soil organic C decomposition by N addition may exceed its stimulating effect on plant productivity, ultimately leading to a net reduction in peatland C storage.

The mechanism of the effect of N addition on CH_4_ emissions is relatively complex, involving the joint regulation of multiple biogeochemical processes [27,28,29,30]. CH_4_ is mainly produced by methanogenic archaea decomposing organic matter under anaerobic conditions and is oxidized by methanotrophic bacteria under aerobic conditions [28]. Nitrate (NO_3_^−^) can effectively inhibit methanogenesis by increasing the redox potential [27,31], while ammonium (NH_4_^+^) can inhibit CH_4_ oxidation by competing with methane-oxidizing bacteria for the activity of methane monooxygenase (MMO), resulting in increased CH_4_ emissions [27,32,33]. In addition, nitrite (NO_2_^−^) produced during nitrification or denitrification has a certain toxicity, which can significantly reduce the activity of methanotrophic bacteria and further weaken CH_4_ oxidation [34,35,36]. Plant functional type plays an important role in the regulation of CH_4_ emissions, especially under N addition conditions. The increase in the proportion of grass plants (such as sedge) may have an important impact on the production and transmission of CH_4_ [30,37,38,39]. The aerenchyma of this type of plant can transport CH_4_ generated in the deep anaerobic environment directly to the atmosphere, bypassing the aerobic zone. At the same time, the root secretions of vascular plants can provide more available substrates for methanogenic archaea, thereby promoting the production of CH_4_ [28,40,41]. In summary, N addition can regulate CH_4_ flux by directly affecting the activity of methanogenic archaea and methanotrophic bacteria, and can also indirectly affect the production and emission of CH_4_ by changing the plant community composition of peatlands. However, this effect may manifest as a promoting effect or an inhibitory effect, and further research is needed to obtain a more comprehensive understanding.

Although previous studies have shown that N addition has an important impact on greenhouse gas fluxes in peatlands, the response mechanism of long-term nitrogen addition (more than 10 years) in temperate peatlands is still unclear. This study was based on a 12-year experimental platform of the Hani peatland in the Changbai Mountains, using an in-situ monitoring method to systematically explore the effects of long-term N addition on CO_2_ and CH_4_ fluxes in peatlands and their potential mechanisms. We hypothesized that: (1) N addition would alleviate nutrient limitations in peatlands, promote the growth of vascular plants, and increase primary productivity (GPP); (2) long-term nitrogen addition would increase the nitrogen content of peatland litter, thereby promoting peat decomposition and increasing ecosystem respiration (ER); (3) the growth-promoting effect of vascular plants induced by nitrogen addition would enhance CH_4_ production and emission through root exudates and aerenchyma, respectively. This study attempts to reveal the regulatory mechanism of long-term nitrogen addition on greenhouse gas fluxes in temperate peatlands and to provide a scientific basis for understanding the carbon cycle dynamics and management of peatlands under the background of global climate change.

## 2. Results

### 2.1. CO_2_ Fluxes

**Figure 1:**
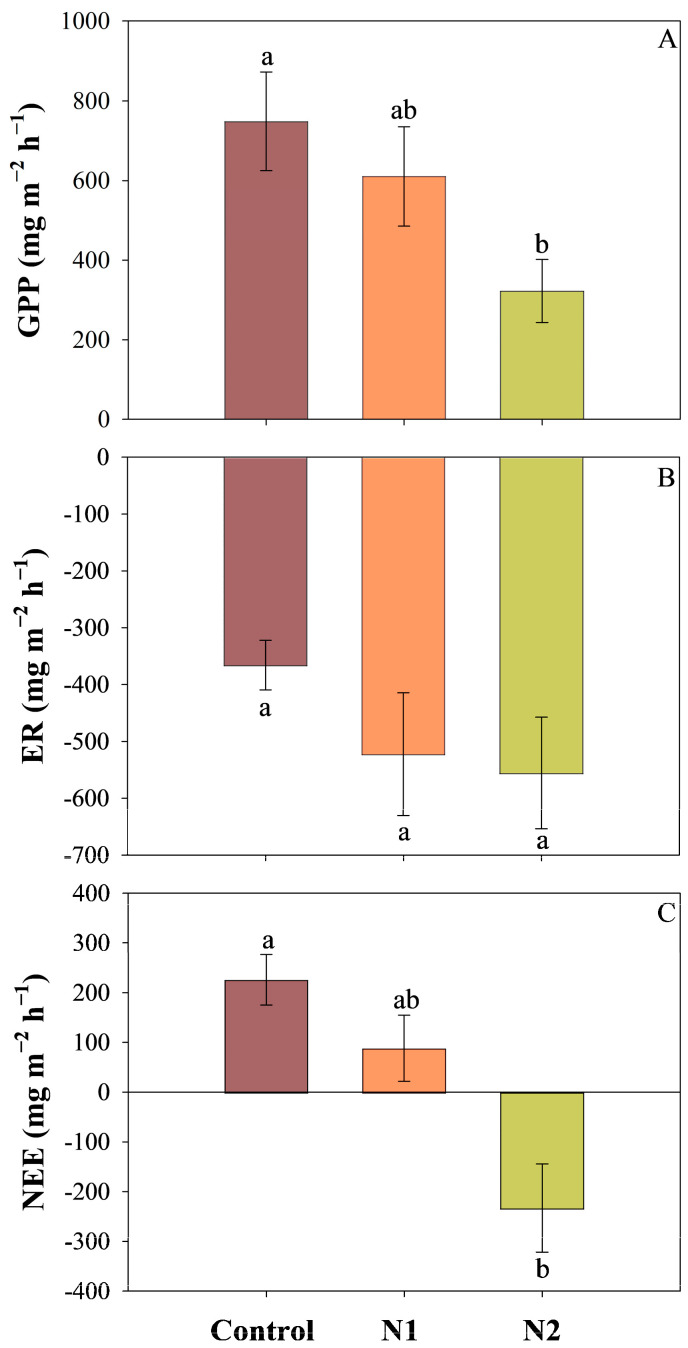
The effects of nitrogen (N) addition (Control: 0 g N m^−2^ yr^−1^, N1: 5 g N m^−2^ yr^−2,^ and N2: 10 g N m^−2^ yr^−2^) on gross primary productivity (GPP, **A**), ecosystem respiration (ER, **B**), and net ecosystem exchange (NEE, **C**) (Mean ± SE, n = 4). Positive values indicate CO_2_ uptake and negative values indicate CO_2_ emission. Different lowercase letters represent significant differences among treatments (Tukey’s HSD test, *p* < 0.05).

Long-term N addition had a significant impact on the gross primary productivity (GPP) (*p* < 0.05) and net ecosystem exchange (NEE) (*p* < 0.01) (Table 1). As N addition increased, both GPP and NEE exhibited a declining trend, especially the N2 treatment significantly reduced the GPP and NEE of the peatland. Compared to the control group, GPP decreased by approximately 56.9% (Figure 1A), while NEE declined by approximately 203.0%, ultimately shifting the peatland from a carbon sink to a carbon source (Figure 1C). Although long-term N addition did not show a significant effect on the respiration rate (ER) (Table 1), ER still showed an increase with higher N addition. Specifically, ER increased by approximately 42.8% and 51.9% under the N1 and N2 treatments, respectively, compared to the control group (Figure 1B).

**Figure 2:**
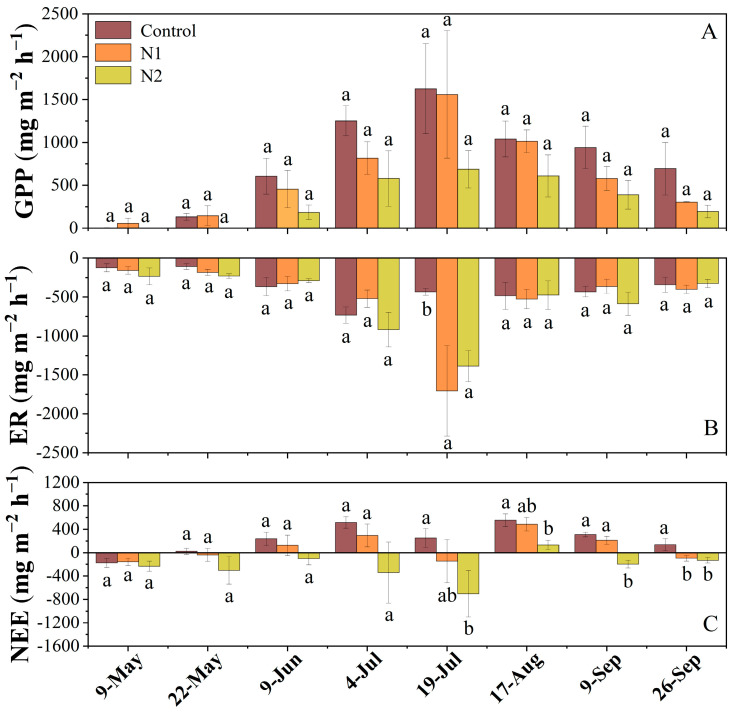
The effects of N addition (Control: 0 g N m^−2^ yr^−1^, N1: 5 g N m^−2^ yr^−1,^ and N2: 10 g N m^−2^ yr^−1^) on the monthly average gross primary productivity (GPP, **A**), ecosystem respiration (ER, **B**), and net ecosystem exchange (NEE, **C**) (Mean ± SE, n = 4) of the peatland in the 2019 growing season. Positive values indicate CO_2_ uptake and negative values indicate CO_2_ emission. Different lowercase letters represent significant differences among the different treatments (Tukey’s HSD test, *p* < 0.05).

Regarding the monthly average variation in GPP, the data on May 9 showed that the GPP under the N1 treatment reached approximately 58.93 mg m^−2^ h^−1^, which was significantly higher than the GPP in the control group (3.23 mg m^−2^ h^−1^, *p* < 0.05) and N2 treatment (2.27 mg m^−2^ h^−1^, *p* < 0.05). This suggests that the N1 treatment advanced the onset of peatland plant photosynthesis. In contrast, the data on May 22 showed that the GPP under the N2 treatment still remained at approximately 2.27 mg m^−2^ h^−1^, which was significantly lower than that of the control (134.50 mg m^−2^ h^−1^, *p* < 0.05), indicating that the N2 treatment delayed the onset of peatland plant photosynthesis. Throughout the growing season, GPP in the N2 treatment consistently remained lower than that in both the control and N1 treatment groups, with the control group exhibiting the highest GPP among all treatments. Notably, GPP in the peatland peaked in mid-July, which is consistent with the typical growing season pattern (Figure 2A).

Similar to the seasonal variation of GPP, the ER in the peatland reached its maximum value during the peak growing season (July), with ER under N addition treatments being significantly higher than that of the control group (*p* < 0.001). Compared to the control, ER increased by approximately 294.0% under the N1 treatment and 220.8% under the N2 treatment (Figure 2B). Regarding the NEE, the peatland functioned as a net C source during the early growing season (May). However, starting in June, the control group and N1 treatment plots generally acted as C sinks, whereas the N2 treatment plots remained a C source throughout the entire growing season (Figure 2C), with this effect being particularly pronounced in July.

### 2.2. CH_4_ Fluxes

**Figure 3:**
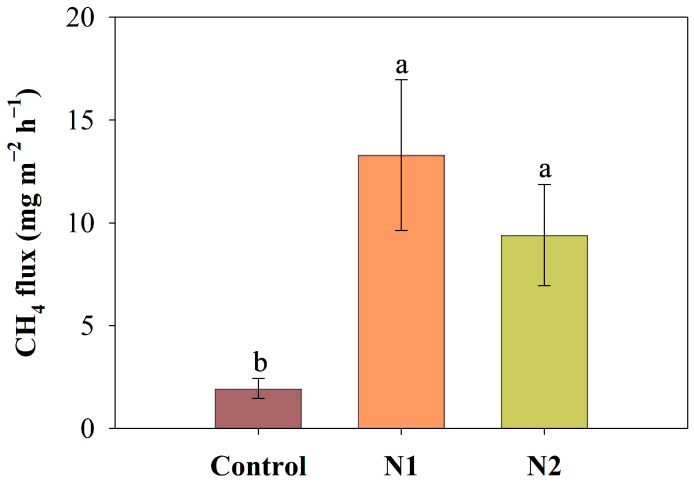
The effects of N addition (Control: 0 g N m^−2^ yr^−1^, N1: 5 g N m^−2^ yr^−1^, and N2: 10 g N m^−2^ yr^−1^) on CH_4_ fluxes (Mean ± SE, n = 4) of the peatland. Different lowercase letters represent significant differences among the different treatments (Tukey’s HSD test, *p* < 0.05).

Long-term N addition significantly affected CH_4_ flux in the peatland (*p* = 0.009, Table 1). Both N1 and N2 treatments significantly increased the CH_4_ emission flux. Compared to the control group, CH_4_ emissions in the N1 and N2 treatments were approximately six times and four times higher, respectively, with the N1 treatment exhibiting the highest average CH_4_ emission flux (Figure 3).

**Figure 4:**
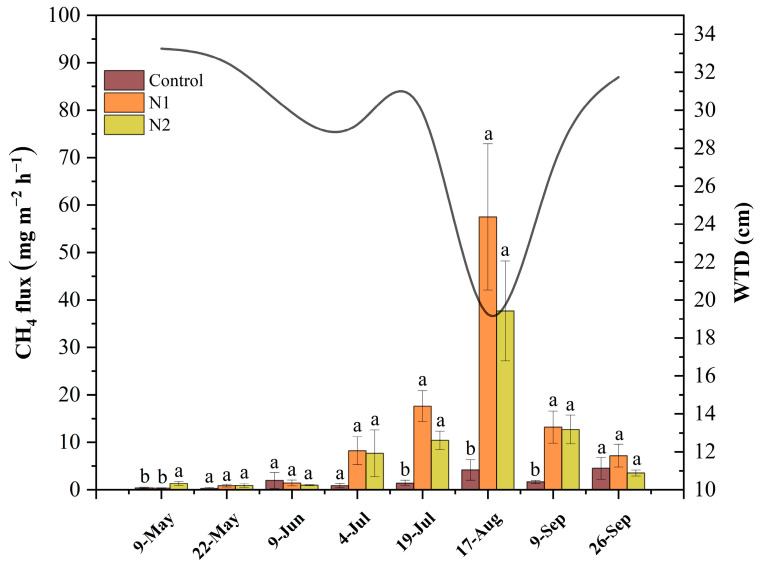
The effects of nitrogen (N) addition (Control: 0 g N m^−2^ yr^−1^, N1: 5 g N m^−2^ yr^−1^, and N2: 10 g N m^−2^ yr^−1^) on monthly average change of CH_4_ fluxes and water table depth (WTD) (black line) in the peatland during the 2019 growing season. The WTD data are from the control group. Different lowercase letters represent significant differences among the different treatments (Tukey’s HSD test, *p* < 0.05).

According to the monthly average variation of CH_4_ flux (Figure 4), the CH_4_ emission flux in the control group remained at a relatively low level throughout the growing season, particularly during the peak growing months (July, August, and September), significantly lower than that in the N addition treatment groups. Unlike CO_2_ flux, CH_4_ emissions in the peatland peaked in mid-August, with the N1 treatment exhibiting the highest CH_4_ flux, approximately 10 times that of the control group. The variation in the CH_4_ flux was closely related to the changes in the water table depth, reaching its highest value (57 mg m^−2^ h^−1^) when the water table depth was at its lowest value (19 cm).

### 2.3. Vegetation Cover

**Figure 5:**
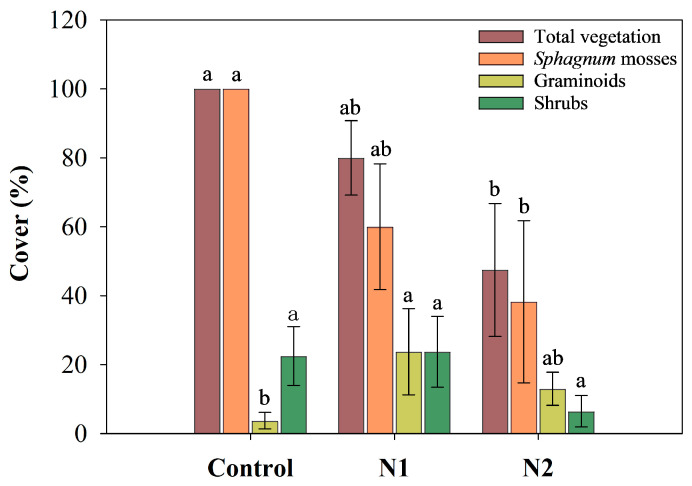
The effects of N addition (Control: 0 g N m^−2^ yr^−1^, N1: 5 g N m^−2^ yr^−1^, and N2: 10 g N m^−2^ yr^−1^) on vegetation cover (Mean ± SE, n = 4). Different lowercase letters represent significant differences among different treatments for the same plant functional type (Tukey’s HSD test, *p* < 0.05).

Long-term N addition had a significant impact on the total vegetation cover of the peatland (*p* = 0.049) (Table 1 and Figure 5). As N addition increased, the total vegetation cover exhibited a declining trend, particularly in the N2 treatment, where it was significantly reduced by approximately 53% compared to the control group. The primary reason for this decline was the reduction in *Sphagnum* moss cover. Although long-term N addition did not show a significant effect on *Sphagnum* moss cover, it still exhibited a decreasing trend with increasing N input, with a reduction of approximately 61.75% compared to the control group. In contrast to the changes in *Sphagnum* moss cover, long-term N addition significantly increased the cover of graminoid in the peatland. The highest graminoid cover was observed in the N1 treatment, where it increased by approximately 20% compared to the control group. The impact of long-term N addition on shrub cover was relatively minor, but in the N2 treatment, shrub cover decreased by approximately 16%.

### 2.4. Environmental Factors and Soil Physicochemical Properties

Overall, long-term N addition significantly affected soil temperature at a depth of 5 cm, water table depth (WTD), and soil moisture (SM) (Table 2). Compared to the control group, WTD in the N1 and N2 treatments decreased by approximately 16.94 cm and 14.1 cm, respectively, while SM increased by approximately 25.18% and 22.67%, respectively. Regarding soil temperature, only the N1 treatment significantly reduced the soil temperature at a depth of 5 cm, decreasing it by approximately 3.1 °C.

In terms of soil physicochemical properties, long-term N addition had a significant impact only on the soil C: N ratio and DOC concentration. The C: N ratio in the control group was approximately 34.04, whereas it decreased to 24.87 and 22.15 in the N1 and N2 treatments, respectively. Additionally, the DOC concentration significantly increased with the N1 and N2 treatments, reaching approximately twice the DOC concentration of the control group (Table 2).

### 2.5. Extracellular Enzyme Activity Potentials

**Figure 6:**
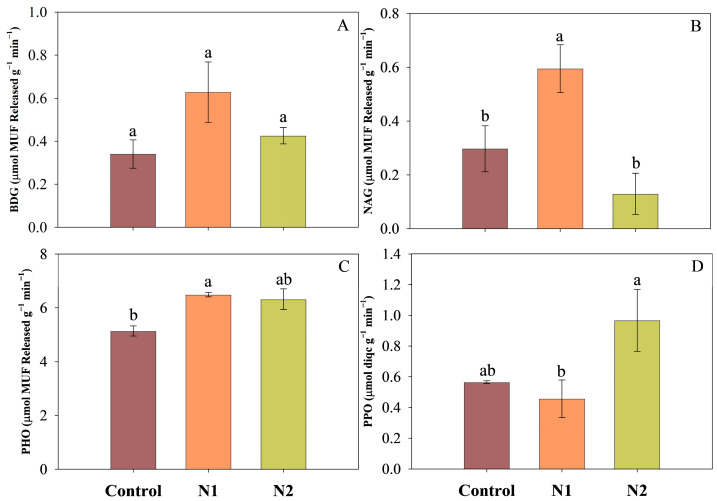
The effects of N addition (Control: 0 g N m^−2^ yr^−1^, N1: 5 g N m^−2^ yr^−1^, and N2: 10 g N m^−2^ yr^−1^) on β-D-glucosidase (BDG, **A**), N-acetyl-β-glucosaminidase (NAG, **B**), phosphatase (PHO, **C**), and phenol oxidase (PPO, **D**) activities (Mean ± SE). Different lowercase letters represent significant differences among the different treatments (Tukey’s HSD test, *p* < 0.05).

Overall, the effects of N addition on β-D-glucosidase (BDG), N-acetyl-β-glucosaminidase (NAG), and phosphatase (PHO) activity potentials followed a similar trend (Figure 6A–C). The N1 treatment significantly enhanced the activity potentials of NAG and PHO, while the increase in BDG activity potentials did not reach statistical significance. Additionally, the N2 treatment significantly increased the activity potentials of polyphenol oxidase (PPO), with its activity reaching approximately 1.7 times that of the control group (Figure 6D).

### 2.6. Correlation Analysis

**Figure 7:**
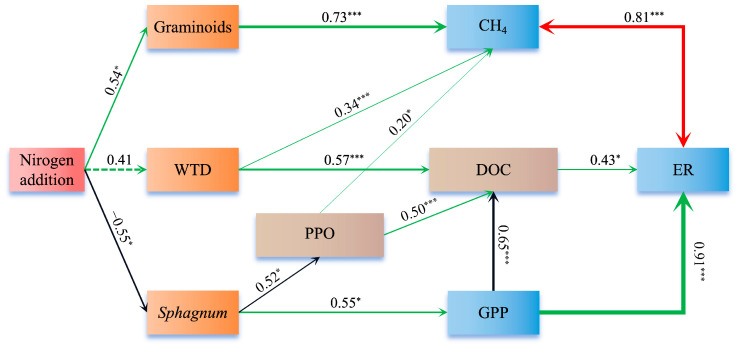
Structural equation model considering all plausible pathways of direct and indirect effects of nitrogen addition on the graminoid cover, *Sphagnum* moss cover, water table depth (WTD), Phenol oxidase enzyme (POX) activity potentials, dissolved organic carbon (DOC) concentration, and Greenhouse gases (CH_4_, GPP, and ER). The numbers on the arrows are the standardized direct path coefficients. The thickness of the arrows represents the size of the path coefficients. The green arrows represent positive effects, the black arrows represent negative effects, the dashed arrows represent negative correlation paths, and the red double arrows represent interaction effects. ‘*’and ‘***’ indicate the significant effect at *p* < 0.05 and *p* < 0.00, respectively. (χ^2^ = 12.665, *p* = 0.942, df = 22, GFI = 0.833, CFI = 1.000, RMSEA = 0.000).

**Figure 8:**
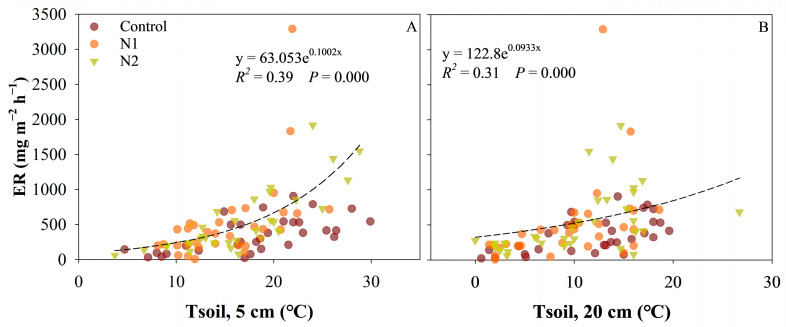
Relationships between the ecosystem respiration (ER) and the soil temperature (Correlation analysis, *p* < 0.05).

The fitting results of the structural equation model indicate that long-term N addition indirectly affects CO_2_ and CH_4_ fluxes by altering the WTD and vegetation growth in peatlands (Figure 7). N addition significantly promoted the growth of graminoid plants while inhibiting the growth of *Sphagnum* mosses. The growth of graminoid plants and *Sphagnum* mosses exhibited significant positive correlations with CH_4_ emissions and GPP, respectively. Long-term N addition also influenced the WTD of the peatland, showing a significant negative correlation with CH_4_ emissions and DOC concentration. Furthermore, DOC concentration was positively influenced by polyphenol oxidase (PPO) activity potential, which was regulated by *Sphagnum* growth—lower *Sphagnum* cover was associated with higher PPO activity potential. ER was affected by DOC concentration and interacted with both CH_4_ emissions and GPP. In addition, ER exhibited a significant positive correlation with soil temperature (Figure 8).

### 2.7. Global Warming Potential

Under natural conditions (Control), peatlands have a cooling effect on the global climate (Table 3). Although peatlands are a source of CH_4_ emissions in their natural state, they absorb a large amount of CO_2_, resulting in a negative net GWP (Table 3). The N1 treatment eliminates the cooling effect of peatlands, primarily due to a significant increase in CH_4_ GWP, leading to a net GWP of 311 g CO_2_ m^−2^ yr^−1^. The N2 treatment raises the net GWP of peatlands to 2169 g CO_2_ m^−2^ yr^−1^, with 71% of this coming from CO_2_ emissions. Overall, N addition eliminates the cooling effect of peatlands and significantly increases their global warming potential.

## 3. Discussion

### 3.1. Effects of Long-Term Nitrogen Addition on CO_2_ Fluxes in Peatlands

Net ecosystem exchange (NEE) is the difference between gross primary production (GPP) and ecosystem respiration (ER), driven by the combined effects of GPP and ER. Long-term N addition (~12 years) led to a decline in NEE, particularly under the N2 treatment, where the peatland’s C sink function disappeared, turning into a C source, primarily driven by the reduction in GPP. The results indicate that long-term N addition significantly reduced GPP and NEE, while its impact on ER was not significant. In the N2 treatment, GPP decreased by more than half compared to the control, which contradicts our initial hypothesis. In this study, the decline in GPP was mainly attributed to the suppression of *Sphagnum* growth by long-term N addition, leading to a reduction in *Sphagnum* cover and consequently decreasing CO_2_ uptake through photosynthesis. Structural equation modeling results showed a significant positive correlation between GPP and *Sphagnum* cover, supporting our explanation for the decline in GPP (Figure 7).

In nutrient-limited peatland ecosystems, short-term N addition can lead to N saturation in the *Sphagnum* layer, causing any excess N to infiltrate into the rhizosphere zone, thereby promoting the growth of vascular plants [31,42]. A comparative study on peat C storage at our research site revealed that N fertilization led to peat decomposition while simultaneously stimulating the growth of vascular plant roots and rhizomes. The increase in underground biomass even compensated for the organic matter loss caused by peat decomposition, ultimately enhancing the entire ecosystem’s C sink function [43]. However, there is still a lack of long-term simulation experiments to assess the profound impacts of N addition on the C sink function of peatlands. Nevertheless, N fertilization over a timescale of approximately a decade has been widely documented to cause excessive NH_4_^+^ accumulation, leading to the production of toxic compounds that inhibit plant growth and ultimately result in *Sphagnum* mortality [7,14,31,44]. Therefore, it is evident that long-term N addition suppresses *Sphagnum* growth, significantly reducing its contribution to peatland C storage. Given that more than half of the global peat originates from *Sphagnum*-dominated peatlands [15], the role of peatlands in the global carbon pool could be severely weakened.

The increase in ER is also one of the factors driving the significant decline in NEE. The current study found that the long-term N addition showed a nonsignificant increase in ER. Previous studies have demonstrated that under high atmospheric N deposition, ER can increase significantly [12,44,45]. Nitrogen addition can enhance N availability in nutrient-poor peatlands, alleviating microbial N limitation and stimulating organic matter decomposition by reducing the C:N ratio of peatland litter [12,46,47,48,49]. Furthermore, phenolic compounds play a crucial role in inhibiting microbial enzyme activity [50]. However, N addition can enhance PPO activity [12], potentially reducing phenolic compound concentrations. This reduction may, in turn, stimulate microbial activity and subsequently increase CO_2_ emissions. In this study, long-term N addition significantly reduced soil C: N and increased PPO activity. As PPO activity increases, complex organic matter is broken down into simpler, more water-soluble organic compounds [51,52], leading to a rise in DOC concentration. Since DOC is an important C source for microbes, its increased concentration can enhance microbial activity [53,54], thereby increasing ER. However, in this study, we did not find a significant rise in ER with increasing N addition. This may be due to the substantial decline in GPP. We found that ER in this study was highly correlated with GPP. A substantial decline in GPP indicates reduced photosynthesis, which subsequently lowers the input of organic C into the soil and ecosystem [55,56], restricting both microbial heterotrophic respiration and plant autotrophic respiration. Ultimately, this dynamic counterbalances the increase in ER driven by the enhanced peat decomposition resulting from N addition. Therefore, long-term N addition primarily regulates CO_2_ fluxes in peatlands by affecting vegetation dynamics.

### 3.2. Effects of Long-Term Nitrogen Addition on CH_4_ Fluxes in Peatlands

Extensive research has demonstrated that N fertilization influences both CH_4_ production and consumption, but the extent and direction of this effect vary widely [32,57]. Some studies have demonstrated that N addition could enhance CH_4_ emissions [20], whereas others have found no significant effect [58] or even a suppressive effect on CH_4_ emissions [13]. In this study, we found that long-term N addition significantly increased CH_4_ flux, aligning with our third hypothesis. Several mechanisms may contribute to the enhancement of CH_4_ emissions by N addition. First, NH_4_^+^ serves as an N source that promotes the growth of methanogenic archaea, thereby increasing methane production. Second, NH_4_^+^ may inhibit CH_4_ oxidation by competing with methane-oxidizing bacteria for methane monooxygenase [20,28,29], leading to elevated CH_4_ emissions. Additionally, the application of NH_4_NO_3_ fertilizer can induce nitrification or denitrification processes, resulting in the accumulation of nitrite (NO_2_^−^), which is toxic and may suppress methane-oxidizing bacterial activity, further reducing CH_4_ oxidation [36].

N addition also indirectly affects CH_4_ production and transportation by altering peatland vegetation composition [28,30,39]. After 12 years of N addition, graminoid cover increased significantly. Moreover, a significant positive correlation was observed between graminoid cover and CH_4_ flux, with the highest values for both occurring in the N1 treatment, further emphasizing the regulatory role of graminoid growth in peatland CH_4_ emissions. Graminoids absorb C through photosynthesis and allocate a portion of it to methanogenic archaea via root exudates, thereby stimulating CH_4_ production [28,40]. Additionally, graminoids and other emergent macrophytes possess aerenchyma tissues, which facilitate CH_4_ transport to the atmosphere, reducing the opportunity for CH_4_ oxidation in the aerobic layer and further increasing CH_4_ emissions [37,38,59]. Thus, long-term N addition may drive a shift in peatland vegetation composition, from *Sphagnum* mosses- to vascular plants-dominated communities. This transformation not only enhances deep CH_4_ production but also suppresses CH_4_ oxidation and increases CH_4_ transport efficiency, ultimately leading to higher overall CH_4_ emissions.

Our study revealed a significant positive correlation between CH_4_ emissions and water table depth (WTD). WTD is one of the key factors determining CH_4_ emissions [28,60,61]. Long-term N addition led to a significant reduction in WTD, meaning the distance between the soil surface and the water surface decreased. Field in situ observations show that this reduction in WTD is primarily due to accelerated peat decomposition, inhibited *Sphagnum* growth, and even its mortality, ultimately resulting in a decline in hummock height. CH_4_ is primarily produced by methanogenic archaea under anaerobic conditions, while a portion of it is oxidized by methanotrophic bacteria in aerobic environments [29]. Consequently, a thicker potential CH_4_ production zone and a thinner oxidation zone led to higher CH_4_ emissions into the atmosphere [60,61,62]. This implies the increase of CH_4_ emissions as the surface-to-water distance decreases. Additionally, under low water table depth conditions, the solubility of CH_4_ in soil water rises. As CH_4_ accumulates in soil pores, it can eventually reach a critical concentration, forming bubbles that rapidly escape to the atmosphere through ebullition [63,64].

## 4. Materials and Methods

### 4.1. Study Site and Experimental Design

Our experiment was established at a transitional mire located in the Changbai Mountains, northeastern China (42°13′ N, 126°31′ E), which has a temperate continental mountain humid monsoon climate with a mean annual temperature of 2.3–3.6 °C and a mean annual precipitation of 757–930 mm [65]. The study site is a near-pristine mire without land-use history. The mean pH of this peatland was 5.4, and the mean peat depth was 4 m. The dominant vegetation in the study area consists of *Sphagnum* mosses (S. imbricatum Hornsch. ex Russow, S. magellanicum Brid., S. flexuosum Dozy & Molk., and S. subsecundum Nees.), dwarf shrubs (Betula ovalifolia Rupr., Vaccinium uliginosum L., Potentilla fruticosa L., and Rhododendron tomentosum Harmaja.), graminoids (Carex lasiocarpa Ehrh., Eriophorum polystachion L., and Phragmites australis (Clav.) Trin.), and the forbs (Smilacina japonica A. Gray and Sanguisorba parviflora (Maxim.) Takeda) [65].

Since the summer of 2007, we applied ammonium nitrate (NH_4_NO_3_) monthly from May to September to simulate elevated N deposition. A total of 12 plots (0.8 m × 0.8 m) were established on separate hummocks dominated by *Sphagnum* in the open area of the peatland. A randomized plot design was used with four replicates and three treatments including control (0 g N m^−2^ y^−1^), N1 (5 g N m^−2^ y^−1^), and N2 (10 g N m^−2^ y^−1^). The amount of N addition was about 2 and 4 times the average level of N deposition in the Changbai Mountains of northeastern China, respectively [66]. Ammonium nitrate was dissolved in distilled water and evenly poured onto the nitrogen addition plots. The unfertilized control plots were watered with the same amounts (300 mL per plot) of distilled water at the same time. The buffer zone between the plots was about 2 m. The plots were connected by a boardwalk system to avoid peat becoming compressed when walking or standing, and to avoid affecting the movement of greenhouse gases during gas sampling.

### 4.2. Measuring Greenhouse Gas Fluxes and Environmental Parameters

Greenhouse gas fluxes were measured twice a month from May to September in 2019, following 12 years of continuous N fertilization. A PVC collar (14 cm high, 25 cm in diameter) with a groove for chamber placement was inserted into each plot to a depth of 5 cm in October 2017. After two years, any root damage and peat disturbance caused by the collar installation should have been restored. Greenhouse gas fluxes were measured on a sunny day from 10:00 to 15:00 local time using a portable greenhouse gas analyzer (LGR-915-0011, Los Gatos Research Inc, San Jose, CA, USA), which was connected to a transparent or a dark chamber (25 cm in diameter and 50 cm in height), fitted into the groove of the PVC collar. The dark chamber blocked light from entering, preventing photosynthesis during the measurement. As a result, the CO_2_ flux measured by the dark chamber represented ecosystem respiration (ER). In contrast, light could penetrate the transparent chamber, allowing photosynthesis to occur during the measurement. Consequently, the CO_2_ flux measured by the transparent chamber represented net ecosystem CO_2_ exchange (NEE). There was only about a 1-min break between measurements from the transparent chamber (for NEE) and the dark chamber (for ER). Therefore, it was reasonable and accurate to estimate gross primary productivity (GPP) based on the NEE and ER measurements taken at the same plot [67]. To ensure an airtight connection with the chamber, each PVC collar was supplied with water sealing in the groove during the measurements. A fan was fixed on top of the chambers to mix and cool down the air inside. The aluminum foil was used to cover the whole chamber exterior to reflect sunlight for heat reduction for the dark chamber. 180 consecutive values of CO_2_ concentration were recorded at 1 Hz time frequency during each 3-min measurement period. The chamber was ventilated for approximately 1 min after each measurement to ensure that the CO_2_ concentration returned to ambient levels before beginning the next measurement. CH_4_ flux and ER were measured simultaneously. For the specific calculation method of greenhouse gas flux, please refer to another article by the author [68].

During each gas sampling campaign, soil temperatures at 5 and 20 cm below the moss surface were measured using a Delta TRAK digital thermometer, while the soil moisture (SM) was assessed with a Stevens moisture meter (TZS-1K, Zhejiang Top Cloud-Agri Technology Co., Ltd., Hangzhou, China) in each plot. Additionally, the photosynthetically active radiation (PAR) in each plot was measured using an Apogee quantum meter (MQ-200, Apogee Inc., Logan, UT, USA) simultaneously with the NEE sampling. NEE was accepted when PAR ≥ 1000 μmol photons m^−2^ s^−1^. The water table depth (WTD) was also measured in PVC tubes installed at each plot during NEE measurements.

The vegetation and plant cover within each collar were assessed by visually estimating the percentage cover of graminoids, shrubs, and *Sphagnum* mosses based on their vertical projection area. All the estimations were carried out by the same observer to minimize between-observer errors. To assure accuracy, vertical photographs of each collar were taken from 20 cm above the ground, and vegetation cover estimates were corrected in the laboratory if necessary.

Furthermore, peat pore water from a depth of approximately 20 cm in each sampling plot was collected to measure dissolved organic carbon (DOC). The DOC concentration was analyzed using a TOC analyzer (TOC-LCPH/TN, Shimadzu Inc., Kyoto, Japan). Before measurement, the peat pore water was filtered using an oil-free diaphragm vacuum pump and a 0.45 μm micro-filtration membrane. Soil samples were collected at the end of July 2019, prior to fertilization and in the absence of recent precipitation, to avoid confounding effects from nutrient leaching caused by heavy rainfall. Soil total carbon (TC) and total nitrogen (TN) were analyzed with an elemental analyzer (Euro Vector 3000, EuroVector S.p.A., Pavia, Italy). Soil total phosphorus (TP) was determined calorimetrically using the ammonium molybdate-ascorbic acid method [69] on a continuous flow analyzer (San + +, Skalar Analytical B.V., Breda, The Netherlands).

### 4.3. Extracellular Enzyme Activity Potentials

The activity potentials of extracellular hydrolytic enzymes were determined using 96-well microplates and fluorescence-based microplate analysis, following the method described by Saiya-Cork et al. (2002) [70]. The activity potentials of the hydrolases β-D-glucosidase (BDG), N-acetyl-β-glucosaminidase (NAG), phosphatase (PHO), and the oxidative enzyme phenol oxidase (PPO) were analyzed with a multifunctional microplate reader (Cytation 5, BioTek). The specific methodology for determining extracellular enzyme activity potentials is detailed in a related study by the author [68].

### 4.4. Statistical Analysis

Data were checked for normality using the residual plots method and were log-transformed when necessary prior to analysis. The effects of N addition on GPP, ER, NEE, and CH_4_ fluxes were examined using repeated measures ANOVA, with the sampling date nested within the sampling block as a random effect. The effects of N addition on vegetation, TC, TN, TP, DOC, N: P ratio, C:N ratio, enzyme activities in the water/soil samples, and other abiotic environmental factors including soil temperature, SM, and WTD, were analyzed using one-way ANOVA. Whenever ANOVA yielded significant effects, Tukey’s HSD test was performed to assess the differences between the control, N1, and N2 treatments. A linear regression model was applied to analyze the relationship between CO_2_ fluxes and the soil temperature. All the statistical analyses above were carried out in R 3.5.3 (https://www.r-project.org/ (accessed on 30 August 2024), R Development Core Team 2019). Statistical significance was accepted at *p* < 0.05.

The structural equation model (SEM) was used to examine the direct and indirect effects of global changes on GPP, ER, and CH_4_, and it was performed in AMOS 22.0 (IBM SPSS, Chicago, IL, USA). SEM was based on the overall dataset. Predicted causal relationships between the variables were based upon prior knowledge, theory, and past experience on the role of N addition and vegetation composition change in peatland CO_2_ and CH_4_ fluxes. The adequacy of model fitting was determined by several tests, including the χ^2^ test (*p* > 0.05, CMIN/df < 2), the goodness of fit (GFI) (values > 0.8), the root square mean error of approximation (RMSEA) (values < 0.05), and the comparative fit index (CFI) (values > 0.9).

Global Warming Potential (GWP) is calculated based on the emissions of CO_2_ and CH_4_. In this study, we used the GWP values recommended in the Sixth Assessment Report (AR6) of the Intergovernmental Panel on Climate Change (IPCC), where the 100-year time horizon GWP values for CO_2_ and CH_4_ are 1 and 32, respectively. The GWP of a gas is calculated using the following formula [71]:

(1) $${GWP}_{{CO}_{2}}=\frac{{CO}_{2}\left(g\;C\;m^{-2}{yr}^{-1}\right)}{MR}\times 1$$

(2) $${GWP}_{{CH}_{4}}=\frac{{CH}_{4}\left(g\;C\;m^{-2}{yr}^{-1}\right)}{MR}\times 32$$

where the ${GWP}_{{CO}_{2}}$ and ${GWP}_{{CH}_{4}}$ represent the GWP of CO_2_ and CH_4_ emissions (g C m^−2^ yr^−1^), respectively. MR is the molar ratio, where the molar ratio for calculating ${GWP}_{{CO}_{2}}$ is 12/44, and for calculating ${GWP}_{{CH}_{4}}$, it is 12/16. The values 1 and 32 represent the GWP of CO_2_ and CH_4_ on a 100-year time scale, respectively. A positive GWP indicates that the peatland is a source of greenhouse gases and can increase the global greenhouse effect; a negative GWP indicates that the peatland is a sink of greenhouse gases and can mitigate the global greenhouse effect.

## 5. Conclusions

By conducting in situ monitoring of CO_2_ and CH_4_ fluxes in a temperate peatland subjected to 12 years of simulated N deposition, this study reveals the effects of long-term N addition on peatland C fluxes and their underlying mechanisms. The results demonstrate that the long-term N addition significantly reduces NEE, shifting the peatland from a C sink to a C source. This shift is primarily driven by a decline in aboveground plant productivity. Over the 12-year N addition period, *Sphagnum* growth was suppressed, even leading to mortality, while N addition promoted the growth of graminoids. However, the increase in graminoid growth did not fully compensate for the decline in GPP caused by *Sphagnum* loss. Instead, it further enhanced CH_4_ emissions from the peatland. Considering the combined effects of long-term N addition on CO_2_ and CH_4_ fluxes, our study suggests that sustained N input may weaken the C sequestration function of northern peatlands, potentially transforming them into C sources and significantly reducing their global cooling effect.

## Figures and Tables

**Table 1:** The effects of nitrogen (N) addition on the total vegetation cover (To-C), *Sphagnum* cover (*Sp*-C), graminoid cover (Gr-C), shrub cover (Sh-C), ecosystem respiration (ER), net ecosystem exchange (NEE), gross primary productivity (GPP), methane (CH_4_) flux, β-D-glucosidase (BDG), N-acetyl-β-glucosaminidase (NAG), phosphatase (PHO), and phenol oxidase (PPO) activities.

Vegetation Cover	*F*	*p*	Greenhouse Gas	*F*	*p*	Enzyme Activity	*F*	*p*
To-C	4.302	0.049	GPP	3.594	0.031	PHO	3.297	0.090
*Sp*-C	3.314	0.083	ER	1.482	0.233	NAG	6.388	0.026
Gr-C	1.628	0.249	NEE	10.996	0.000	BDG	1.750	0.228
Sh-C	1.392	0.297	CH_4_	4.913	0.009	PPO	3.894	0.060

**Table 2:** The effects of N addition (Control: 0 g N m^−2^ yr^−1^, N1: 5 g N m^−2^ yr^−1^, and N2: 10 g N m^−2^ yr^−1^) on soil temperature at 5 cm (Tsoil, 5 cm) and 20 cm (Tsoil, 20 cm) below the moss surface, water table depth (WTD), and soil moisture (SM) (Mean ± SE, n = 4). Different lowercase letters represent significant differences among the different treatments (Tukey’s HSD test, *p* < 0.05).

Treatment	T_soil_, 5 cm (°C)	T_soil_, 20 cm (°C)	WTD (cm)	SM (%)	TC (%)	TN (%)	TP (g/kg)	C:N Ratio	N:P Ratio	DOC (mg/L)
Control	18.25 ± 1.14 ^a^	11.72 ± 0.97 ^a^	29.83 ± 1.36 ^a^	9.93 ± 0.88 ^b^	37.79 ± 0.33 ^a^	1.11 ± 0.03 ^b^	0.54 ± 0.04 ^a^	34.04 ± 0.96 ^a^	20.78 ± 1.37 ^a^	3.71 ± 0.47 ^b^
N1	15.15 ± 0.81 ^b^	9.62 ± 0.90 ^a^	12.89 ± 0.88 ^b^	37.47 ± 4.72 ^a^	35.11 ±2.15 ^a^	1.43 ± 0.14 ^ab^	0.53 ± 0.06 ^a^	24.87 ± 1.25 ^b^	27.27 ± 1.66 ^a^	7.54 ± 1.48 ^a^
N2	16.53 ± 1.06 ^a^	10.67 ± 1.06 ^a^	15.73 ± 0.76 ^b^	32.60 ± 4.37 ^a^	32.96 ± 1.79 ^a^	1.50 ± 0.06 ^a^	0.61 ± 0.09 ^a^	22.15 ± 1.85 ^b^	25.90 ± 2.56 ^a^	7.29 ± 0.79 ^a^

**Table 3:** The average global warming potential (GWP) of the emitted greenhouse gases (CO_2_ and CH_4_) under the different treatments (Control: 0 g N m^−2^ yr^−1^, N1: 5 g N m^−2^ yr^−1^, and N2: 10 g N m^−2^ yr^−1^) during the 2019 growing season.

Treatments	GWP (g CO_2_ m^−2^ yr^−1^)		
	CO_2_	CH_4_	Net GWP
Control	−1492	130	−1361
N1	−582 (0.0)	893 (100.0)	311
N2	1537 (71)	632 (29)	2169

Positive values represent warming and negative values represent cooling. The numbers in brackets indicate the contribution of each greenhouse gas to the net GWP.

## Data Availability

Data will be made available on request.
